# Design and Additive Manufacturing of a Continuous Servo Pneumatic Actuator

**DOI:** 10.3390/mi14081622

**Published:** 2023-08-17

**Authors:** Gabriel Dämmer, Hartmut Bauer, Michael Lackner, Rüdiger Neumann, Alexander Hildebrandt, Zoltán Major

**Affiliations:** 1 Institute of Polymer Product Engineering, Johannes Kepler University Linz, 4040 Linz, Austriazoltan.major@jku.at (Z.M.); 2 Advanced Development Control and Robotics, Festo SE & Co. KG, 73734 Esslingen, Germany

**Keywords:** pneumatic actuator, additive manufacturing, soft robotics, elastomer molding, control of soft actuators

## Abstract

Despite an emerging interest in soft and rigid pneumatic lightweight robots, the pneumatic rotary actuators available to date either are unsuitable for servo pneumatic applications or provide a limited angular range. This study describes the functional principle, design, and manufacturing of a servo pneumatic rotary actuator that is suitable for continuous rotary motion and positioning. It contains nine radially arranged linear bellows actuators with rollers that push forward a cam profile. Proportional valves and a rotary encoder are used to control the bellows pressures in relation to the rotation angle. Introducing freely programmable servo pneumatic commutation increases versatility and allows the number of mechanical components to be reduced in comparison to state-of-the-art designs. The actuator presented is designed to be manufacturable using a combination of standard components, selective laser sintering, elastomer molding with novel multi-part cores and basic tools. Having a diameter of 110 mm and a width of 41 mm, our prototype weighs less than 500 g, produces a torque of 0.53 Nm at 1 bar pressure and a static positioning accuracy of 0.31° with no limit of angular motion. By providing a description of design, basic kinematic equations, manufacturing techniques, and a proof of concept, we enable the reader to envision and explore future applications.

## 1. Introduction

### 1.1. Motivation

#### 1.1.1. Lightweight Robotics

Lightweight robots are manipulators for cage-free operation in temporary environments, such as flexible production lines and medical assistance situations. A quest for particularly lightweight, compact, and affordable systems with compliant and back-drivable behavior [1] has given rise to a variety of actuation concepts. Pneumatic actuators have attracted interest because of their simple design, low weight, and low cost, and functional prototypes of pneumatically actuated robots with articulated joints have already been described [2,3,4]. In soft robotics where different compliant actuation methods, such as electrohydrodynamic devices [5], are researched, the easily manufacturable pneumatic actuators are still the most common [6,7,8].

#### 1.1.2. Pneumatic Devices for Continuous Rotation

The motion range of double-acting rotary pneumatic actuators, such as rotary vane actuators [3,9] and rotary bellows actuators [2,10], is generally less than 360°. Robots using these actuators thus have a limited workspace, and mechanical stops must be bypassed. Further, applications that require a continuous rotation of the robot’s hand axis, such as screwing or polishing, cannot be accomplished. However, some fluidic devices are capable of continuous rotary motion. The underlying functional principle is that three or more linear actuators are sequentially pressurized to continuously or incrementally push forward a rotor. By analogy to electric motors, the sequential and selective pressurization of linear actuators is hereafter referred to as “commutation”.

Mechanical commutation means that the opening and closing of fluidic passageways for sequential pressurization of the linear actuators is realized by rotating mechanical components. Musser [11] described a “fluid wave generator” for strain wave gears (also known as “harmonic drive gears”) in which fluidic cylinder-piston actuators are arranged radially. In the device, a drive shaft contains channels that—as the drive shaft rotates—sequentially connect opposing cylinders to the supply pressure. Lewis [12] claimed to increase the versatility of a similar mechanism by adding a valve to control the back pressure in the cylinders during the venting phase. Stoianovici [13] described another fluidic strain wave generator for a medical stepper motor in which sequential pressurization is realized by the rotation of an external radial piston pump. Cunningham [14] presented a mechanically commutated pneumatic radial piston motor based on the roller-cam principle, commercial versions of which have been developed [15,16]. The drawbacks of mechanical commutation include that a fixed relation exists between the angle of rotation and the opening cross-sections of the fluidic passageways, and thus the timing and duration of the pressure pulses as well as the actual pressure trajectory of the individual pulses cannot be actively controlled. Furthermore, mechanical commutation requires precise mechanical components for dynamic sealing.

In solenoid valve commutation, an electrical circuit or controller is used to sequentially open and close solenoid valves connected to the linear actuators of the rotary device. This commutation approach has been used in pneumatic stepper motors. Cissell et al. [17] described a pneumatic wave generator of a harmonic drive gear that features opposing pairs of diaphragm actuators, with each pair being connected to a solenoid valve. Suzumori et al. [18] reported on another solenoid-commutated stepper motor, in which multiple elastomeric chambers are sequentially pressurized. Stoianovici et al. [19] presented a pneumatic stepper motor for medical robots, which was later patented [20], and experimented with mechanical and solenoid commutation. Groenhuis and Stramigioli [21] described a piston-driven pneumatic stepper motor with solenoid commutation. Gong et al. [22] reported on solenoid commutation in the context of soft rotary actuators that use the sequential inflation of elastomeric bladders to rotate either a toothed drive shaft or an external rotor. Rizk et al. [23] invented a rotary joint that contains a fluidic strain wave actuator with diaphragm cylinders and employs an annular band with a friction surface instead of the typically used toothed interface. In contrast to mechanical commutation, the use of solenoid valves and electronics allows the timing and duration of the pressure pulses to be modified, although this has not yet been mentioned explicitly. However, the pressure trajectories, that is, the actual courses of chamber pressures, cannot be modified. Creating smooth torque curves or torque trajectories is therefore not possible.

#### 1.1.3. Prototyping of Bellows Actuators

Additive manufacturing (AM) enables quick and cost-efficient iterations of design, manufacturing, and testing, and can facilitate the transition from early-stage prototyping to small series production, as demonstrated by Gealy et al. [1]. AM is particularly suited to the manufacturing of integrated fluidic systems and could become key to the long-term success of pneumatic lightweight robots. Soft inflatable actuators, such as bellows actuators, are most suitable for AM-based prototyping because they require neither tight dimensional tolerances nor smooth surfaces to function properly [24,25,26]. Many examples of soft inflatable actuators have already been presented, derived mainly from soft robotics [27,28,29,30,31], and various AM-based manufacturing procedures have been described. Recent AM-based approaches differ primarily in whether the soft components are manufactured directly from an elastomeric material, or by using rigid molds that are manufactured first and from which the final parts are obtained in a second step [32]. In an early approach, Peele et al. [33] used digital mask-projection stereolithography to manufacture micro bellows actuators that enable a variety of bending motions. More recent publications have demonstrated the use of PolyJet technology for the manufacturing of bellows actuators [34,35]. However, while PolyJet and stereolithography impose few constraints on the complexity of the structures produced and typically require at most two manufacturing steps (printing and removal of support material), the photocurable materials used exhibit a complex [36] and comparatively inferior mechanical behavior and can withstand only relatively small strains, especially if subjected to repeated loadings [35,36,37,38]. In a recent publication, Sparrmann et al. [32] demonstrated the use of Rapid Liquid Printing (RLP)—a novel, silicone-based process—for the manufacturing of soft bellows actuators. The bellows actuators obtained were clearly superior to PolyJet-printed versions in terms of mechanical performance, but the RLP technology has not yet been commercialized and is therefore not widely accessible. In the absence of accessible, simple, and cost-efficient methods for the direct AM of resilient elastomer bellows, researchers have developed various molding processes. The fundamental advantage of this indirect approach is that a variety of inexpensive but mechanically highly resilient materials can be processed, such as silicone and polyurethane (PU) elastomers. However, to produce highly complex parts, measures must be taken to enable the demolding of undercuts in the molded part. Common strategies involve using soluble core and mold materials [32,39,40,41,42] or soft cores that can be removed after curing [43,44]. However, these techniques complicate and prolong the manufacturing process, and additional waste is produced in most cases.

### 1.2. Contribution, Originality, and Organization of the Paper

**Figure 1:**
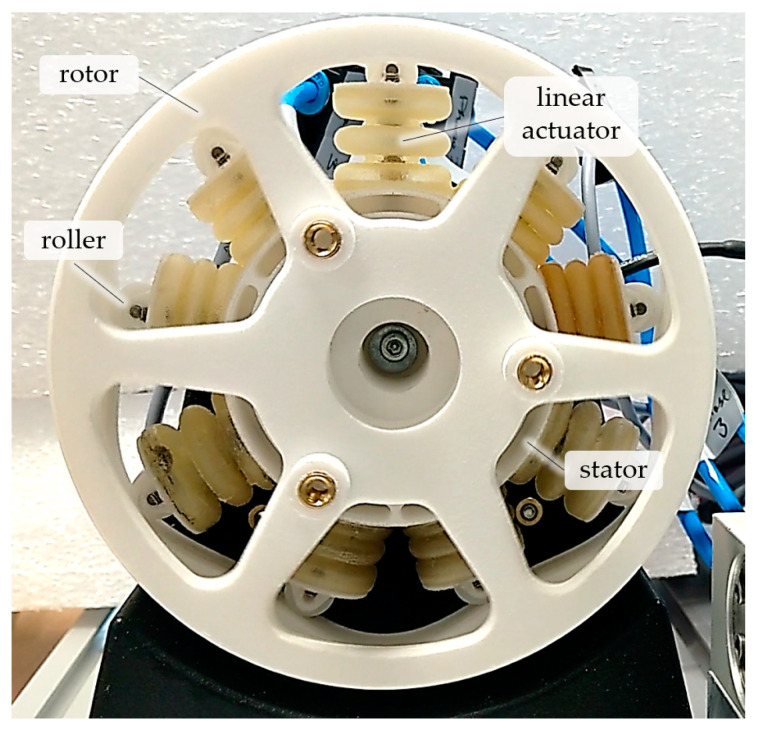
Servo pneumatic actuator for continuous rotation and positioning. Rotor and stator were obtained directly via selective laser sintering of polyamide powder. The polyurethane elastomer bellows structures were molded using laser-sintered molds.

Additively manufactured pneumatic robots could develop into a competitive approach for future lightweight robots, with low cost and inherent compliance. However, typical pneumatic actuators have an inherently limited motion range, and pneumatic devices that are capable of continuous rotary motion have hitherto been unsuitable for servo pneumatic applications. The research described here addresses these issues by presenting a servo pneumatic actuator that is capable of continuous rotation and includes a rotary encoder, pressure sensors, and proportional valves to enable independent closed-loop control of the phase pressures and actuator position. It is demonstrated that the novel actuator concept is not only functional but that the smoothness of rotation can indeed be altered by the choice of pressure profiles, and is not fixed as with previous designs. The prototype actuator (see Figure 1)—which we consider to be the first of its kind—was manufactured largely using AM and is complemented with simple standard components to facilitate replicability. Novel additively manufactured three-part molding cores are presented, which allow for the manufacturing of complex-shaped bellows structures while minimizing waste. This article provides a starting point for understanding the fundamental kinematics, establishing a suitable manufacturing strategy, and performing initial tests and validation experiments. The kinematic relations and the prototyping techniques developed are shown in detail and may inspire further research in this field. The remainder of this paper is organized as follows: First, the operating principle is explained in general terms. An implementation of this operating principle which is particularly suitable for AM is then presented, and the manufacturing steps required are detailed. Finally, experiments that validate the functioning of the prototype actuator are described.

## 2. Operating Principle

### 2.1. Conceptual Design

**Figure 2:**
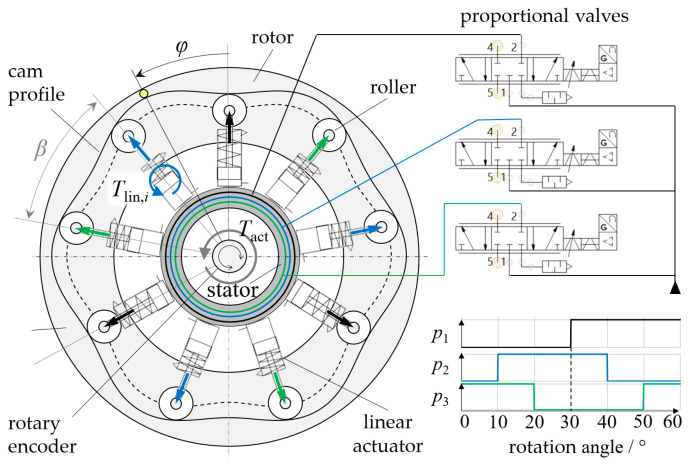
Schematic illustration of our novel rotary actuator. Each linear actuator exerts a radially directed force that causes a reaction torque *T*_*lin*,*i*_ by means of a roller and the cam profile. The rotary actuator torque *T_act_* is the sum of the torques *T*_*lin*,*i*_ produced by the individual linear actuators. The angles *φ*, *β*, and *β*_off_ are the rotation angle of the actuator, the angle between two adjacent linear actuators, and the angle between adjacent actuators of the same pressure phase, respectively. Pressure phases are indicated by black, blue and green arrows and lines. Depending on the pressure trajectories used, the actuator can be employed for a variety of purposes, such as continuous rotation, positioning, and braking. In the simplest case, rectangular pressure trajectories are applied, and phase pressures are shifted according to the phase offsets, as shown in the diagram.

This section describes, in general terms, the operating principle of the rotary actuator, a schematic view of which is shown in Figure 2. The main components are a circular rotor with a periodically curved inward-facing cam profile, various radially arranged linear single-acting fluidic actuators with rollers that are in in contact with the cam profile, a stator holding the linear actuators, a device for measuring the rotation angle, and various pneumatic proportional valves. When pressurized, each linear actuator exerts a radially directed force that is transmitted by a roller to the cam profile of the rotor. Depending on the slope of the cam profile at the point of contact with the roller, torque is created. The linear actuators are grouped into phases of equal pressure, and each phase pressure *p_i_* (*i* = 1…*n*) is controlled independently using a proportional valve. Applying a suitable, angle-dependent pressurization scheme (commutation) causes the continuous rotation of the rotary actuator. While the use of multiple linear actuators per phase (*a* ≥ 1) is optional, a minimum of three phases is required to enable rotation in both directions and from any starting position.

In the example shown, nine linear actuators are equally spaced around the circumference, which results in the actuators being offset by 40° relative to each other (*β* = 40°). As the actuators are grouped into three phases (*i* = {1, 2, 3}), the number of actuators per pressure phase is *a* = 3, and linear actuators of the same phase are offset by 120°. The rotation angle *φ* of the actuator is defined as the angle between the rotor that contains the cam profile and the stator that contains the linear actuators. To achieve a clockwise continuous rotation, the actuator torque must be positive in every angular position. This can be achieved using a simple sequence of rectangular pressure trajectories where pressure is applied only when the slope of the cam profile in the point of contact is negative (see Figure 2 for an example). The torque generated by the complete rotary actuator *T_act_* is the sum of the torques of linear actuators *T*_*lin*,*i*_ per phase times the number of actuators *a* per phase:

(1) $$T_{act}(\varphi ,\;p_{i})=a\cdot \sum_{i=1}^{n}T_{lin,i}\left(\varphi ,\;p_{i}\right),$$

and the derivation will be described below.

### 2.2. Actuator Torque

**Figure 3:**
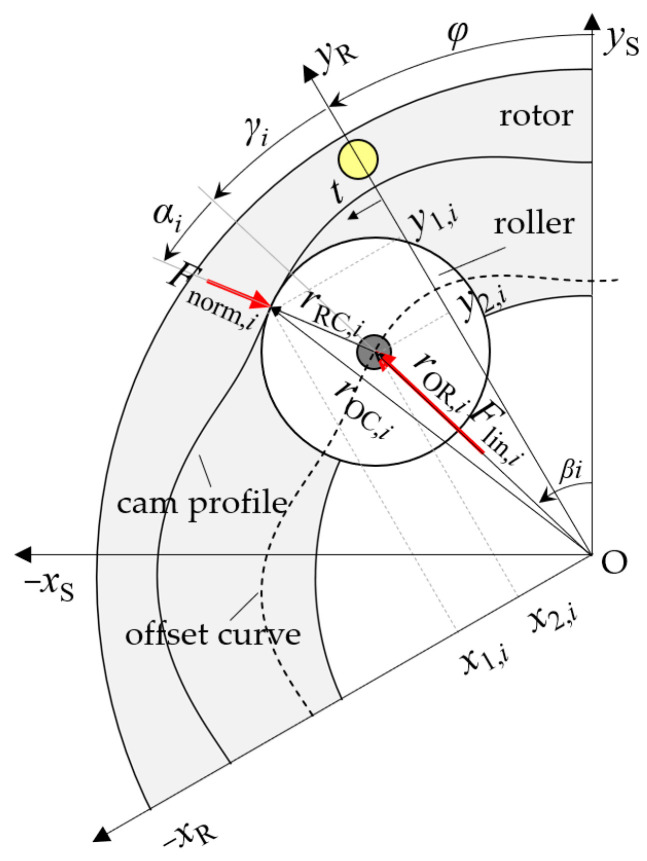
Kinematic relations and forces of a roller. Two cartesian coordinate systems are drawn, where the “S-system” with the coordinate axes *x*_S_, *y*_S_ is fixed to the stator and the “R-system” with the coordinate axes *x*_R_, *y*_R_ is fixed to the rotor. The angles *φ*, *β_i_*, and *γ_i_* are the rotation angle of the rotor, the position of the linear actuator, and their difference, respectively. The pressure angle *α_i_* is between the radially directed force *F*_*lin*,*i*_ and the reaction force component *F*_*norm*,*i*_ normal to the cam profile. The coordinates of the cam profile (*x*_1_, *y*_1_) and offset curve (*x*_2_, *y*_2_) are described as functions of *t* in the “R-system”. The position vectors ${\vec{r}}_{OR,i}$, ${\vec{r}}_{OC,i}$ and ${\vec{r}}_{RC,i}$ describe the point of contact, the roller center point, and their connection, respectively.

Figure 3 shows a single roller as it is in contact with the cam profile of the rotor, and position vectors, angles, and forces are indicated. If the phase offset angle between adjacent linear actuators within a phase is an integer multiple of the cam’s profile’s period, it is sufficient to investigate the kinematic relations for a single linear actuator of each phase *i*.

The static equilibrium of forces for the described system yields (without derivation) the blocked torque exerted by a single linear actuator of the phase *i*:

(2) $$T_{lin,i}\left(\gamma_{i},p_{i}\right)=F_{lin,i}(p_{i})\cdot \tan\left(\alpha_{i}(\gamma_{i})\right)\cdot \left|{\vec{r}}_{OR,i}(\gamma_{i})\right|,$$

where $F_{lin,i}(p_{i})$ is the radially directed force of the linear actuator, $\alpha_{i}(\gamma_{i})$ is the pressure angle, and $\left|{\vec{r}}_{OR,i}(\gamma_{i})\right|$ is the amount of the roller center point’s coordinates. That is, the factor $\tan\left(\alpha_{i}(\gamma_{i})\right)$ defines the relation of the exerted radial force and tangential reaction force component, and $\left|{\vec{r}}_{OR,i}(\gamma_{i})\right|$ is the effective lever arm of that tangential reaction force component.

The phase-specific pressure angle $\alpha_{i}(\gamma_{i})$ can be calculated using the position vectors ${\vec{r}}_{OR,i}\left(\gamma_{i}\right)$ and ${\vec{r}}_{RC,i}(\gamma_{i})$ as (for simplicity, the dependence on *φ_i_* is omitted hereafter)

(3) $$\alpha_{i}=\arccos\left(\frac{{\vec{r}}_{OR,i}\;\cdot \;{\vec{r}}_{RC,i}}{\left|{\vec{r}}_{OR,i}\right|\;\cdot \;\left|{\vec{r}}_{RC,i}\right|}\right).$$

Relative to the rotor, the roller center point follows a curve that is offset from the cam profile by the radius of the roller. We hereafter refer to this curve as the “offset curve”. Given that the cam profile is described by a parametrized function with the parameter *t* and the differentiable elements (*f*_1_(*t*), *f*_2_(*t*)) = (*x*_1_(*t*), *y*_1_(*t*)), the offset curve (*x*_2_(*t*), *y*_2_(*t*)) is

(4) $$\left(\begin{array}{c} x_{2}(t)\\ y_{2}(t) \end{array}\right)=\left(\begin{array}{c} x_{1}(t)\\ y_{1}(t) \end{array}\right)+r\cdot {\vec{n}}_{t}=\left(\begin{array}{c} x_{1}\left(t\right)+\frac{r\cdot \;y_{1}^{’}\left(t\right)}{\sqrt{{{(x}_{1}’(t))}^{2}+{{(y}_{1}’(t))}^{2}}}\\ y_{1}\left(t\right)-\frac{r\;\cdot x_{1}^{’}\left(t\right)}{\sqrt{{{(x}_{1}’(t))}^{2}+{{(y}_{1}’(t))}^{2}}} \end{array}\right),$$

where *r* is the offset, and ${\vec{n}}_{t}$ is the unit normal vector of the initial curve at the point of contact. Using Equation (4) and the phase-specific rotation angle $\gamma_{i}$ (0 ≤ $\gamma_{i}$ *≤* 2π) as the parameter, the position vectors ${\vec{r}}_{OR,i}(\gamma_{i})$, ${\vec{r}}_{OC,i}(\gamma_{i})$ and ${\vec{r}}_{RC,i}(\gamma_{i})$, shown in Figure 3, can be calculated. The cam profile expressed as a function of $\gamma_{i}$ (*f*_1_($\gamma_{i}$), *f*_2_($\gamma_{i}$)) = (*x*_1_($\gamma_{i}$), *y*_1_($\gamma_{i}$)) equals the contact point’s position vector ${\vec{r}}_{OC,i}\left(\gamma_{i}\right)$ (Equation (5)); the position vector of the roller center ${\vec{r}}_{OR,i}\left(\gamma_{i}\right)$ is defined by the offset curve values (Equation (6)); and the connection vector ${\vec{r}}_{RC,i}(\gamma_{i})$ is the difference between ${\vec{r}}_{OC,i}\left(\gamma_{i}\right)$ and ${\vec{r}}_{OR,i}\left(\gamma_{i}\right)$ (Equation (7)):

(5) $${\vec{r}}_{OC,i}\left(\gamma_{i}\right)=\left(\begin{array}{c} x_{1}\left(\gamma_{i}\right)\\ y_{1}\left(\gamma_{i}\right) \end{array}\right);$$

(6) $${\vec{r}}_{OR,i}\left(\gamma_{i}\right)=\left(\begin{array}{c} x_{2,i}(\gamma_{i})\\ y_{2,i}(\gamma_{i}) \end{array}\right);$$

(7) $${\vec{r}}_{RC,i}\left(\gamma_{i}\right)=\left(\begin{array}{c} x_{2,i}\left(\gamma_{i}\right)\\ y_{2,i}\left(\gamma_{i}\right) \end{array}\right)-\left(\begin{array}{c} x_{1,i}\left(\gamma_{i}\right)\\ y_{1,i}\left(\gamma_{i}\right) \end{array}\right).$$

By inserting the given equations backwards and using the relation $\gamma_{i}=\beta_{i}-\varphi$ for conversion between the phase-specific rotation angle and the actuator rotation angle, the actuator torque $T_{act}(\varphi ,\;p_{i})$ can be calculated for any given design and phase pressures. The cam profile of the actuator described in this paper is given in polar coordinates:

(8) $$r\left(\gamma_{1}\right)=50\;mm+2.5\;mm\cdot \cos\;\left(6\gamma_{1}\right).$$

Thus, the cam profile has an amplitude of 2.5 mm and a period of 60° and is radially offset by 50 mm. To calculate the offset curve and phase torques, a parametric expression in Cartesian coordinates can be obtained using

(9) $$x_{1}\left(\gamma_{1}\right)=r\cdot \cos\left(\gamma_{1}\right);y_{1}\left(\gamma_{1}\right)=r\cdot \sin\left(\gamma_{1}\right).$$

The radial force *F*_*lin*,*i*_ acting on each roller of a phase *i* is a function of the phase pressure *p_i_* and depends on the effective cross-sectional area *A* of the linear actuator well as frictional forces *F_fric_* and structural forces *F_struct_*:

(10) $$F_{lin,i}=p_{i}\cdot A-F_{fric}-F_{struct}.$$

**Figure 4:**
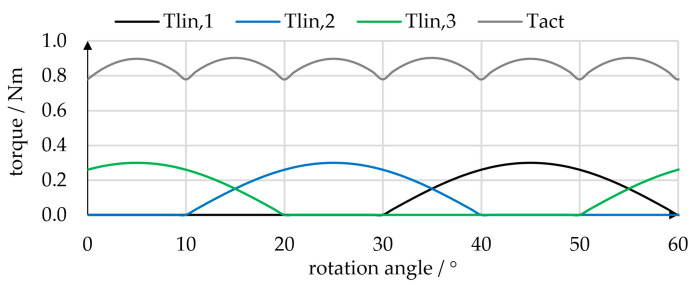
Superposition of torques. The rotary actuator torque *T_act_* is three times the sum of the torques *T*_*lin*,1_, *T*_*lin*,2_ and *T*_*lin*,3_ created by the individual linear actuators. In this example, rectangular pressure trajectories of 1 bar and a cosinusoidal cam profile were used.

The linear actuators used have an inner diameter of 16 mm, which gives an effective cross-sectional area of *A* = 201 mm^2^. Omitting frictional and structural forces of the bellows yields $F_{lin,i}$ = 20.1 N/bar for the force generated by each linear actuator. Using Equation (4) allows the offset curve to be computed, and the pressure angles can be obtained from Equation (3) with Equations (6) and (7). Computing the torques created by the individual linear actuators (Equation (2)) and the total torque (Equation (1)) for the pressure trajectories given in Figure 2 yields the torque profiles, as shown in Figure 4. The linear actuator torques are 60° periodic and comprise 30° intervals of positive torque and 30° intervals of zero torque. The linear actuator torques are shifted by 40°, and, according to Equation (1), the total torque is the sum of the linear actuator torques multiplied by three. Peaks in the total torque occur at the linear actuator torque maximum and at each intersection point, that is, every 10°. Since there is no change in sign of the total torque, the simple rectangular pressure trajectories seem suitable for achieving continuous rotary motion of our prototype actuator. However, the actual torque was expected to be lower, as no frictional effects or structural forces of the bellows were considered.

## 3. Design and Manufacturing

To test the previously described actuation concept, a prototype actuator was developed. This section comprises (A) a description of the linear actuators, including material modeling, FE-based design, and an AM-based molding technique, and (B) descriptions of the rigid actuator parts and of the complete prototype.

### 3.1. Linear Actuators

Bellows actuators are especially suitable for AM-based prototyping, without the need for high-precision tools and CNC (computer numerical control) machines, and we developed linear bellows actuators for the servo pneumatic rotary actuator described in this paper. The soft bellows structures can be manufactured using laser-sintered molds with an addition-curing polyurethane (PU) elastomer of Shore 80 A hardness [45]. The PU has an elongation at break of 750% and excellent tear strength [45].

#### 3.1.1. Material Model

**Figure 5:**
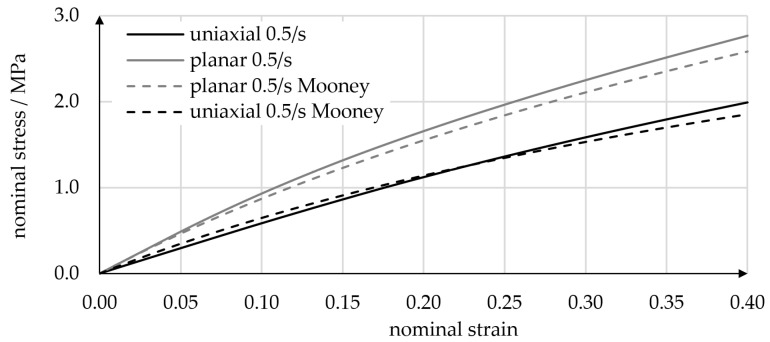
Uniaxial and planar tensile testing of PU specimens for calibration of hyperelastic material models. Nominal strain rates were 0.5 1/s in both planar and uniaxial tests.

We performed (i) uniaxial tensile experiments using standard specimens [46] (type 5A) with a nominal thickness of 2 mm and (ii) planar tensile experiments using rectangular plate specimens with a large aspect ratio of 10 mm/100 mm and a thickness of 1 mm, as described by Çakmak et al. [47]. In the experiments, specimens were elongated at strain rates of 0.5 1/s by means of a linear actuator (Bose Corp., 222 ElectroForce Systems Group, Eden Prairie, MN, USA) and the resulting reaction forces were measured using a WMC-25 load cell (Interface Inc., Scottsdale AZ, USA). All experiments were performed at an ambient temperature of 21 °C. Figure 5 plots the nominal uniaxial and planar tensile stresses over nominal strains. Each curve is an average of three experiments, and each experiment was performed using a pristine specimen. As is typical of elastomers, the stress–strain relations were nonlinear, and planar experiments yielded larger stresses. Several hyperelastic material models were calibrated following the Abaqus internal calibration procedure [48], which uses a non-linear least-squares method to minimize deviations of the model prediction from experimental stress values. A simple Mooney–Rivlin material model yielded good accordance with the tensile experiments while showing stable behavior beyond the fitting range and well into the compression regime. Note that we did not include compression test data, as they are difficult to obtain for this strain rate and include frictional effects. The Mooney–Rivlin model for incompressible materials relates the strain energy potential *U* to the first and second invariants *I*_1_ and *I*_2_ of the left Cauchy–Green deformation tensor:

*U* = *C*_10_(*I*_1_ − 3) + *C*_01_(*I*_2_ − 3), (11)

where *C*_10_ and *C*_01_ are the material parameters that must be calibrated to the experimental data. In Figure 5, experimental results are compared to predictions based on the Mooney–Rivlin model (*C*_10_ = 0.525 MPa; *C*_01_ = 0.723 MPa). Detailed information on the Mooney–Rivlin model and its implementation in finite elements (FE) software can be found in the literature [48,49,50].

#### 3.1.2. Bellows Shape

**Figure 6:**
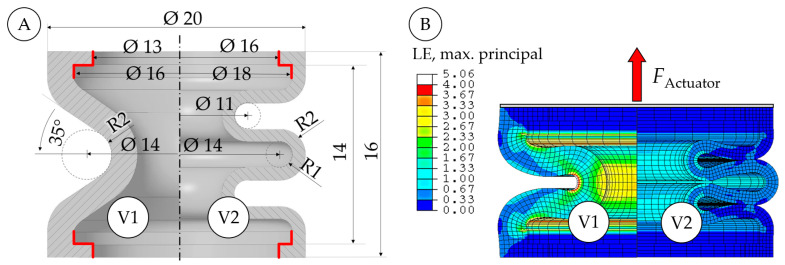
Shape variants (**A**) and FE simulations (**B**) of linear bellows actuators. While the V1 shape can easily be molded, the V2 shape requires much less force to be compressed and structural strains are considerably lower.

In the next step, a suitable bellows shape was defined. Initially, we considered using a simple bellows shape that had previously been described in the context of a material model calibration of a PolyJet elastomer [35]. As discussed below, this simple initial shape was unsuitable, and an improved, more complex shape was required for the rotary actuator described in this paper. In Figure 6A, cross sections and dimensions of the initial (V1) and improved (V2) bellows shapes are shown. Both versions have an outer diameter of 20 mm and a height of 16 mm, and contain mounting lips at the top and the bottom. V1 has a wall thickness of 2 mm and contains only two half-waves without severe undercuts. The small mounting lips feature only minor undercuts and can easily be demolded. In V2, the wall thickness was decreased to 1 mm, and an additional wave was added to reduce structural stiffness. Using the material model described above, FE simulations of both shapes were performed, some results of which are shown in Figure 6B. Pressure loads of 1 bar were applied to the inner surfaces of the bellows, and force loads were added to model the pressure force on the flanges. In the rotary actuator described in this paper, the nine linear bellows actuators perform strokes of 5 mm and alternate continuously between a strain-free state at the top dead center and a compression of 5 mm at the bottom dead center. Figure 6B shows a contour plot of the strain distribution in the bellows structures at 2.5 mm of compression, that is, at the midpoint of the stroke. In V1, maximum principal strains are considerably larger than in V2, and thus reduced fatigue life should be expected. The strain maximum of about 100% in V2 was considered acceptable. In V1, a compression of 2.5 mm caused a reaction force of 32.6 N. Increasing the pressure to 1 bar increased the actuator force to 49.5 N. This means that 66% of the maximum output force results from structural deformation and cannot be modified by air pressure, which makes V1 an unfavorable design for the intended use. V2 requires only 1.5 N to be compressed by 2.5 mm and produces an output force of 15.3 N at 1 bar. Thus, only 10% of the maximum force output is due to structural deformation, while the remaining 90% can be modified via the air pressure applied.

#### 3.1.3. Validation Experiment

**Figure 7:**
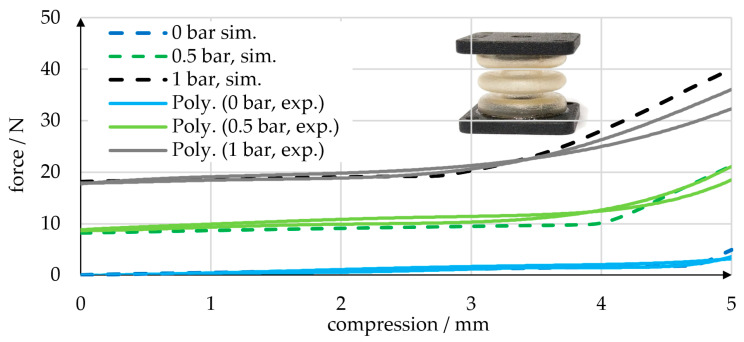
Comparison of experiments (polynomial regressions) and simulations in terms of the displacement-dependent force exerted by a linear bellows actuator with a V2 bellows shape at 0 bar, 0.5 bar, and 1 bar of pressure. A sudden increase in force was caused by self-contact of adjacent bellows waves. Larger pressures caused a stronger inflation of the bellows and led to self-contact at smaller displacements. The small figure shows one of the PU bellows specimens used.

In order to validate the function of the preferable V2, we manufactured the bellows test components shown in Figure 7 (small image). In the validation experiments, displacement cycles with compression between 0 mm and 5 mm were performed at constant pressure levels of 0, 0.5, and 1 bar, and the exerted force was measured. Experiments were performed using the same setup as for the tensile experiments described above, but with a pressure regulator and a manual shut-off valve included. Several thousand load cycles were performed without failure, which proves the general functionality of the V2 bellows. In Figure 7, a representative selection of the experimental results is shown.

The exerted force is clearly dependent on the pressure applied. For small displacements, the force remains relatively constant, showing little and approximately linear dependency on displacement. At larger displacements, a sudden increase in force was observed. At some point, adjacent waves of the bellows structure came into contact with each other—a phenomenon that we refer to as “self-contact”. In the zero-pressure state, self-contact occurred at almost 5 mm of compression. At increased pressures of 0.5 and 1 bar, the inflation of the bellows caused self-contact to occur already at about 4 mm and 3 mm of compression, respectively. We ran FE simulations of the experiments and compared the results. As shown in Figure 7, the simulations are capable of predicting the nearly linear increase in force and self-contact of the bellows structure. Note that the experimental output forces in the non-deflected state were 16% and 27% larger than in the initial simulations. We attribute these deviations to inaccuracies in the air pressures applied and modified the simulated pressure loads accordingly. Two bellows shapes were compared: one designed for easy manufacturability (V1) and the other (V2) with a greater focus on the desired mechanical properties. While the introduction of an additional wave in V2 resulted in more suitable structural behavior with lower stiffness, the severe undercut due to the additional wave requires considerable effort to de-mold. We developed a molding technique that utilizes multi-part cores to enable the manufacturing of the V2 geometry, and the molds, cores, and exact procedures are described below.

#### 3.1.4. Molding of Elastomer Bellows

**Figure 8:**
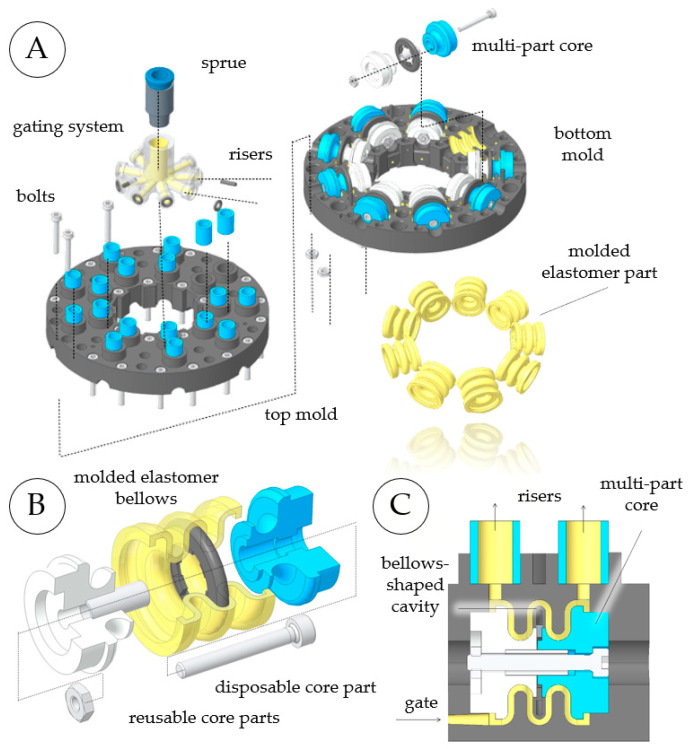
Molding of elastomer bellows structures using a two-part mold with a nine-fold cavity and multi-part cores. The mold assembly consists of top and bottom molds, nine multi-part cores, and a gating system (**A**). The multi-part cores include two reusable parts and a ring that is removed destructively to demold the undercut of the bellows’ middle wave (**B**). The nine cavities are formed by the molds and multi-part cores, with the liquid material entering the cavities at their lowest points (**C**).

The main parts of the mold assembly (see Figure 8) are the top and bottom molds, nine multi-part cores placed between the molds, and a gating system that distributes liquid material from the sprue to the nine bellows cavities. The gating system is affixed to the bottom mold using O-ring seals and metal pins. The molds, cores, and gating system are laser-sintered polyamide 12 (PA12) parts. For the sprue and risers, an 8 mm pneumatic connector and 8 mm pneumatic tube pieces are used, respectively. The assembly is complemented with screws and nuts that connect the top and bottom molds. To achieve the desired compliance in the molded bellows structure, the bellows shape must contain multiple waves. However, the middle wave of the desired bellows shape cannot be demolded when single-part or two-part cores are used. For this reason, we developed novel multi-part cores that enable the manufacture of complex multi-wave bellows. In Figure 8B, a multi-part core is shown together with a single bellows structure. These cores consist of three polymer pieces which are screwed together, and grooves in the core parts and aluminum sleeves ensure radial alignment during molding. Figure 8C shows the assembled multi-part core mounted between the top and bottom molds. The cavity with the desired bellows shape is formed between the outer surface of the core and the inner surfaces of the molds. The molds are connected to the gating system by gates at the lowest points of the cavities, and risers are placed at the highest points. After molding, the outer core parts can easily be extracted because they have only minor undercuts. The middle core part is a hollow ring that fills in the severe undercut of the middle wave and must be removed destructively using needle-nose pliers. As the outer core parts are completely reusable, only 0.2 g of PA12 material per core is lost in each molding process. The liquid PU precursors are processed at ambient temperature and polymerize via a polyaddition reaction without relevant shrinkage. To prevent adhesion and facilitate demolding, a release agent and sealing agent were applied before each and every fifth batch, respectively. PU precursors were mixed in accordance with the data sheet [45], using polymer cups and stainless-steel stirring rods, and degassed at 0.1 bar in a vacuum chamber (absolute pressure) for several minutes. The material was poured into a syringe and pressed through the sprue into the gating system and the nine bellows cavities. The molds containing the liquid material were then pressurized at 6 bar (relative pressure) for several hours. Liquid urethanes absorb atmospheric moisture, which causes the formation of CO_2_ bubbles. Curing at an elevated pressure collapses existing gas bubbles and avoids the formation of new ones. As the PU precursors contain potentially hazardous toluene diisocyanate [51], materials were processed under an extractor hood, and gloves and safety glasses were worn. We chose SLS for mold-making, as it offers sufficient geometrical accuracy and relatively smooth surfaces. However, for the molding of smaller objects with wall thicknesses below 1 mm, or if even smoother surfaces are required, three-dimensional (3D) inkjet printing can be used instead. If a lower mechanical performance of the molded parts is acceptable, silicone elastomers can be used, which can be cured at ambient pressure and are less hazardous than the PU used here.

### 3.2. Actuator Prototype

**Figure 9:**
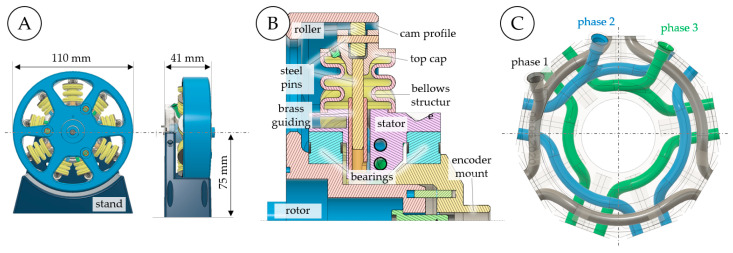
CAD drawings of the novel servo pneumatic rotary actuator with radially arranged linear bellows actuators. Front and side views show the main dimensions (**A**). A sectional view of one of the linear bellows actuators shows the internal components (**B**). The stator has integrated air channels that connect the linear actuators of each phase (**C**).

Using the kinematic design and cam profile described in the Section 2 together with the bellows shape defined in the Section 3, a prototype actuator (see Figure 9) was designed using CAD (computer-aided design). The actuator measures 110 mm in diameter and 41 mm in width. For experimental validation, a stand was attached (Figure 9A), which is not required for use in a robot. As shown in Figure 9B, the rotor is mounted to rotate relative to the stator by means of two polymer ball bearings (BB-16006-B180-10-ES, Igus GmbH, Köln, Germany). Each linear bellows actuator includes a top cap with a PTFE (polytetrafluoroethylene) roller and a steel pin with outer diameters of 2 mm and 9 mm, respectively. The translatory motion of each actuator is guided by another steel pin (3 mm diameter) and a brass insert which are glued into the top cap and stator, respectively. At the top dead center, the overlap between steel pin and brass insert is 13 mm to provide smooth guidance. The stator contains numerous fluidic channels that connect the bellows actuators of each phase, as shown in Figure 9C. This specific channel design is the result of several iterations of design and testing, and is tailored to the SLS manufacturing process. SLS is a powder-based AM method in which certain areas of a powder bed are sintered in a layer-wise manner. This procedure enables the manufacturing of very complex internal channels and overhangs; however, non-sintered powder remains in any hollow structures and must be removed after the build process. As shown in Figure 9C, the channel systems for each phase are subdivided into three sections, each of which connects two adjacent bellows. This design provides easy access to the non-sintered powder. We used channel diameters of 3.1 mm and avoided tight curvatures to better facilitate powder removal. The powder was easily removed by means of steel wire, pipe cleaners, and compressed air.

Before assembly, the brass inserts and 3 mm steel pins shown in Figure 9B were lubricated with silicone-based grease. Instant adhesive was then used to attach the elastomer bellows to the stator and top caps. Bearing pretension can be adjusted by means of a screw that pulls the rotor against the encoder mount. The rotor, stator, stand, top caps, and encoder mount are laser-sintered PA12 parts and can therefore be printed together with the molds described in Section 3. All other parts can be either purchased or manufactured using standard tools. The implementation described here is merely one instance of the actuation concept introduced in this paper. While the design is tailored to SLS manufacturing and intended for initial experiments, it can be altered and modified according to other application requirements. Manufacturing can, for example, be simplified by using (even more) accessible FDM (fused deposition modeling) printers if a lower resolution is acceptable. Silicone actuators can be used to facilitate manufacturing. Independent of the manufacturing technique applied, the design does not need any dynamic seals or mechanical components for commutation. The proportional valves can be placed as required by the application, which makes particularly compact robots conceivable. Unlike in pneumatic RVAs, the design described has no strict requirements in terms of the concentricity of the rotor and stator, and small radial or axial displacements do not restrict its function.

## 4. Experimental Validation

### 4.1. Experimental Setup and Procedure

**Figure 10:**
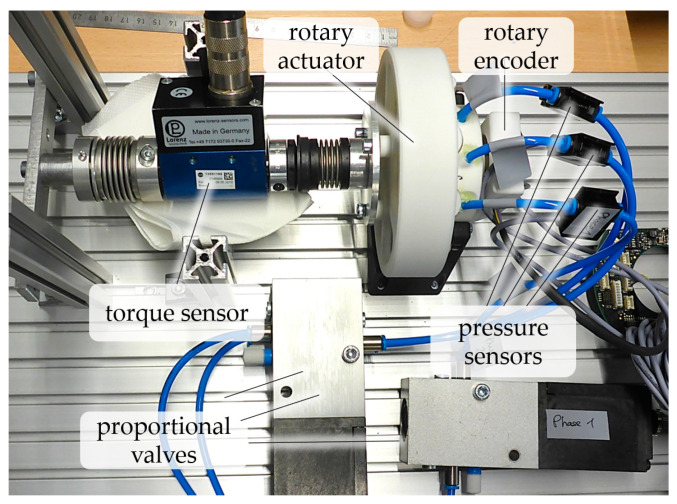
Experimental investigation of a novel AM-based pneumatic actuator for continuous rotation and angular positioning. The actuator is connected to a torque sensor and a rotary encoder, and each of the three actuator phases is connected to a proportional valve and a pressure sensor.

A series of experiments was performed to validate the main functionalities of our actuator, that is, continuous rotation and positioning. The experimental hardware setup is shown in Figure 10. A Heidenhain (Dr. Johannes Heidenhain GmbH, Traunreut, Germany) ECI 11118 rotary encoder and a Lorenz (Lorenz Messtechnik GmbH, Alfdorf, Germany) DR-2112 torque sensor were mounted on the prototype actuator. To control and measure the pressures of the three actuator phases, three Festo MPYE-5-M5-LF (Festo Se. & Co. KG, Esslingen, Germany) proportional valves and three Festo SPTE-P10-R-Q4 pressure sensors were used, respectively. A dSPACE (dSPACE GmbH, Paderborn, Germany) prototyping computer (not shown) with a DS1005 processor card was used to control the valves and capture the sensor data. The processor card was programmed via MATLAB/Simulink (The MathWorks, Inc., Natick, MA, USA). A simple controller was implemented, which comprised proportional position and velocity cascades and a pressure controller with compensation for the valve behavior.

### 4.2. Continuous Rotation

**Figure 11:**
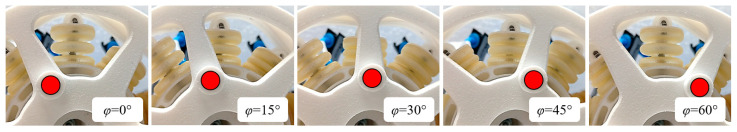
Continuous rotation of the AM-based prototype pneumatic actuator. The sub-panels show individual frames of a videoclip that illustrate the rotary actuator at angular positions between 0° and 60° as the state of each individual linear bellows actuator is 60° periodic (see Figure 4 for reference).

In Figure 11, the prototype actuator is shown in operation. Each subpanel corresponds to a frame from a video clip that shows the continuous rotation of the actuator. The sequence demonstrates the general functionality of the actuator, which results from the conversion of translatory to rotary motion. A video of the actuator in operation is provided as Supplementary Material (Video S1).

**Figure 12:**
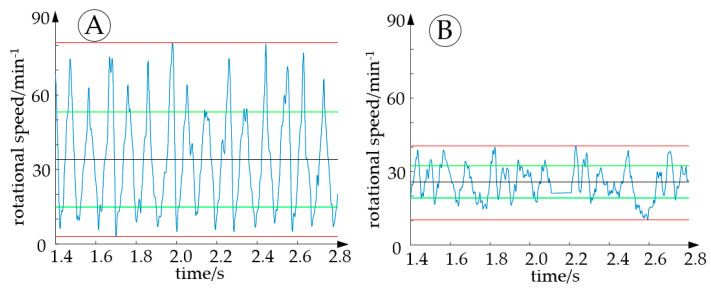
Measurements of rotational speed for two types of pressure trajectories. Using rectangular pressure trajectories resulted in considerable variation in rotational speed (**A**), which was reduced by using “constant-torque” pressure trajectories (**B**). The blue lines indicate the rotational speeds and the red, green, and black lines indicate the extrema, standard deviations, and mean values of the rotational speeds, respectively.

In this case, the rectangular pressure trajectories shown in Figure 2 were applied. The top bellows actuator is pressure-free at angles between 0° and 30°, and pressure is applied at angles between 30° and 60° to push forward the cam profile. While continuous rotation was possible in both directions and from any starting position, the motion appeared relatively jerky and noisy. As shown in Figure 4, the rectangular pressure trajectories create a rough torque curve. Additional tests were performed to investigate whether a smoother motion can be achieved by changing the pressure trajectories. We compared the rotational speeds of the actuator when simple rectangular target pressure trajectories or “constant-torque” pressure trajectories were used. Figure 12 plots the time-dependent rotational speed of the prototype. Applying rectangular pressure trajectories resulted in a mean rotational speed of 34 1/min with a standard deviation of 19.2 1/min. Using the “constant-torque” pressure trajectories, the mean rotational speed was 26.1 1/min with a considerably lower standard deviation of 6.58 1/min. In the “constant torque” pressure trajectories, kinematic relations causing the angular dependency of torque were fully compensated for. Since some effects—especially friction—were not yet considered, torque and rotational speed were not completely constant. However, rotation was markedly smoother, and this initial test demonstrates that the choice of pressure trajectories using proportional valves can influence the behavior of the actuator considerably.

### 4.3. Positioning

**Figure 13:**
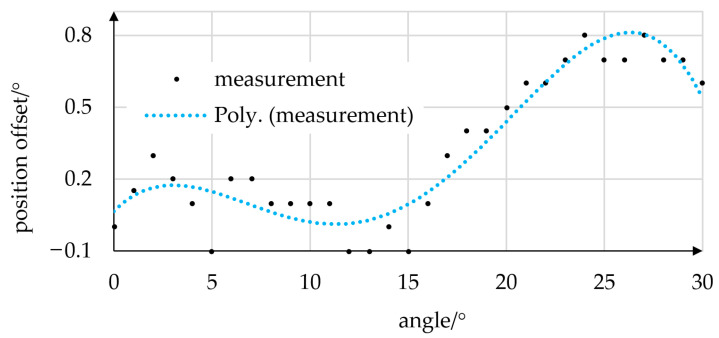
Positioning accuracy of the prototype. Static position offsets were measured at target angles of 0–30°. Offset values (black dots) were approximated by a third-degree polynomial regression curve (blue). A simple proportional position controller was used.

In order to prove the actuator’s positioning capability and investigate the presence of potential stopping points, the actuator was set to target angles between 0° and 30° in 1° steps. Before each measurement, the actuator was set back to the 0° position. Position offsets were then determined from the difference between target and measured values. The offset values, approximated using a third-degree polynomial, are plotted in Figure 13.

The mean offset was 0.31°, with maximum values of 0.8° being reached at 24° and 27°. Given that only a simple proportional position controller was used, we consider the accuracy achieved very promising.

**Figure 14:**
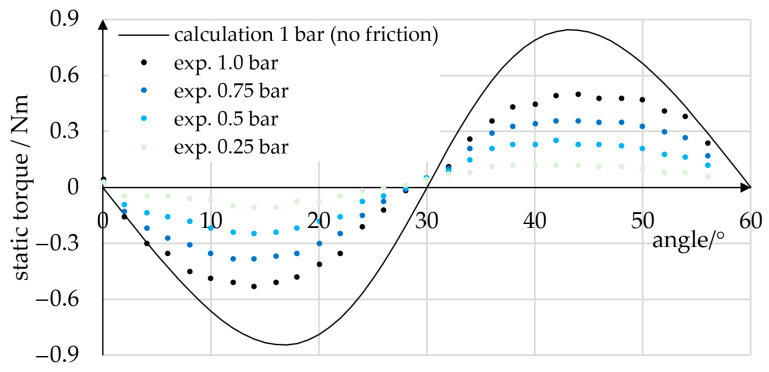
Static torque of the prototype for various pressure levels. The analytical calculation (black graph) predicts the qualitative progression of the actual torque curve (dots). The quantitative deviation between calculation and experiment is attributed mainly to frictional effects.

Static torque measurements at angles between 0° and 60° were performed. During measurement, a constant torque was applied to a single phase, and torque values were recorded in 2° steps. In the procedure, the torque sensor was manually fixed to the prototype, which resulted in an angular position accuracy of about 0.5°. In Figure 14, the static torque values measured are plotted for pressures of 0.25, 0.5, 0.75, and 1.0 bar. For comparison, the frictionless theoretical static torque at 1.0 bar is plotted. At angles of 0°, 30°, and 60°, the tangential vector component of the contact force disappears, and thus the torque passes zero. At 1.0 bar, the absolute theoretical maximum torque is 0.84 Nm, and the measured torque was 0.53 Nm. We attribute this deviation mainly to frictional effects.

### 4.4. Discussion of Experiments

We have demonstrated that the main functions of continuous rotation and positioning can be achieved using the described actuator design in combination with simple rectangular pressure trajectories. This represents a fundamental advance for today’s pneumatic double-acting rotary actuators and mechanically commutated devices. We have also shown that smoother rotation can be achieved by altering the pressure trajectories applied, and thus making use of proportional valves for commutation as an advancement to today’s electrically commutated pneumatic devices. Our results demonstrate that positioning to within accuracies of 0.8° and torques of up to 0.5 Nm can be achieved. Using a better controller would most likely increase positioning accuracy. Our results also indicate that friction reduces the actuator torque considerably, and we therefore intend to model and reduce frictional effects. The derivation and investigation of advanced pressure trajectories and controllers that take into account the frictional effects will be a part of future research. Comparative experiments, based on prototypes of similar dimensions, materials, and manufacturing technologies will be needed to allow for a quantitative comparison of the presented concept to state-of-the-art devices.

## 5. Conclusions

We have described the design, AM, and experimental validation of a novel servo pneumatic actuator for continuous rotation and positioning. Especially designed for AM, our actuator is easily replicable, and design modifications can be realized rapidly. Similar to radial piston engines, our actuator achieves rotary motion by alternating the actuation of radially arranged linear actuators. Our prototype rotary actuator includes nine linear bellows actuators that are equally distributed around its circumference and are grouped into three pressure phases. Each bellows actuator comprises an elastomeric bellows structure, a guiding mechanism, and a top cap with a roller that is in contact with the inward-facing sinusoidal cam profile of the rotor. Pressurization of a pressure phase causes the corresponding bellows actuators to expand, which in turn pushes forward the cam profile. Applying a sequence of suitable pressure trajectories to the three pressure phases causes the rotation of the rotor. In contrast to previous rotary devices that use mechanical parts or solenoid valves, our actuation system includes a rotary encoder, pressure sensors, and proportional valves to enable independent closed-loop control of the phase pressures and to eliminate the need for dynamic seals, which cause friction and cannot be additively manufactured directly.

We used a PU elastomer of Shore 80 A hardness in combination with laser-sintered molds to fabricate the soft bellows structures. The non-linear large-strain behavior of the PU was modeled using a hyperelastic material model that was calibrated to uniaxial and planar tensile test data. FE simulations were performed in order to define a suitable bellows shape, and it was found that molding would require the use of lost cores. In order to minimize material waste, we developed novel three-part cores that include two larger reusable (2.5 g) and one disposable part (0.2 g). To distribute the liquid PU precursors to the nine cavities and minimize the loss of material (6.2 g), a separate, disposable distributor, for which the main mold components (120.8 g) can be reused, was designed. We applied the SLS process to thermoplastic PA12 powder to manufacture the rotor and stator. The stator contains complex internal channels that connect the bellows and valves, and the channel design facilitates the removal of powder residue.

Our prototype has a diameter of 110 mm, a width of 41 mm, and weighs less than 500 g. In initial experiments, the prototype achieved continuous rotation, and we demonstrated that the appropriate choice of pressure trajectories improves the smoothness of rotation. Using a simple position controller, we achieved a static position accuracy of 0.31° and static torques of up to 0.53 Nm at 1 bar pressure. We assume that these values can be improved further by increasing the pressure amplitude and modifying the pressure trajectories. In the context of pneumatic lightweight robotics, our actuator overcomes several problems of state-of-the-art actuators. Continuous rotation enables drilling and polishing or fastening screws to a defined torque or position. This work is an initial step towards pneumatic rotary actuators with freely programmable commutation. Introducing variable commutation has considerably improved the efficiency and performance of internal combustion engines and electric motors, and it is now up to future research to tap into this potential for continuous pneumatic actuators.

## Data Availability

Data are contained within the article or cited articles.

## Supplementary Materials

The following supporting information can be downloaded at https://www.mdpi.com/article/10.3390/mi14081622/s1, Video S1: Dämmer et al. Design and Additive Manufacturing of a Continuous Servo Pneumatic Actuator.
